# Efficacy of combined rigid and flexible ureteroscopy for complex ureteral stones in type 2 diabetes mellitus

**DOI:** 10.3389/fendo.2025.1607275

**Published:** 2025-07-21

**Authors:** Liang Jiang, Shu-Ben Sun, Qi-Long Miao, Ze-Jun Yan

**Affiliations:** Department of Urology, The First Affiliated Hospital of Ningbo University, Haishu, Ningbo, Zhejiang, China

**Keywords:** type 2 diabetes mellitus, complex ureteral stones, combined rigid and flexible ureteroscopy, percutaneous nephrolithotomy, postoperative sepsis

## Abstract

**Background:**

Type 2 Diabetes Mellitus (T2DM) patients with complex ureteral stones face significant challenges in terms of treatment outcomes, including higher risk for complications such as sepsis and prolonged recovery times. The efficacy of combined rigid and flexible ureteroscopy in managing these stones in T2DM patients remains underexplored.

**Methods:**

A retrospective cohort study was conducted at our hospital from January 2021 to August 2024. The study included patients aged 18–65 years diagnosed with T2DM and complex ureteral stones (size >1 cm, multiple stones, or those in difficult-to-reach areas). Exclusion criteria involved uncontrolled urinary tract infections, renal malformations, and other significant comorbidities that could hinder surgical success. A total of 182 patients were included, with 93 receiving combined rigid and flexible ureteroscopy (observation group) and 89 undergoing percutaneous nephrolithotomy (PCNL) (control group). The study followed STROBE guidelines, and ethical approval was obtained. Preoperative blood glucose control and surgical interventions were managed in accordance with standard protocols.

**Results:**

The observation group exhibited superior perioperative outcomes, with significantly shorter surgery time, less intraoperative blood loss, and a reduced duration of hematuria compared to the control group. Stone clearance at 7 days postoperatively was significantly higher in the observation group (54.84%) compared to the control group (39.33%) (P=0.036). Additionally, CRP levels were lower in the observation group at 3 and 5 days postoperatively, indicating less postoperative inflammation. The incidence of postoperative sepsis was significantly associated with female gender, age ≥60, BMI ≥25 kg/m², preoperative positive urine culture, and elevated CRP and fasting plasma glucose (FPG) levels.

**Conclusions:**

Combined rigid and flexible ureteroscopy offers a promising approach for managing complex ureteral stones in T2DM patients, providing better early stone clearance, reduced postoperative complications, and improved recovery outcomes compared to percutaneous nephrolithotomy. Risk factors for postoperative sepsis in this patient population include older age, female gender, higher BMI, preoperative urine culture positivity, and elevated CRP and FPG levels. Further studies are necessary to confirm long-term benefits and optimize sepsis prevention strategies.

## 1 Introduction

Ureteral stones—particularly complex ones—pose a significant clinical challenge in urology due to their variability in location, size, and composition. Effective management often requires advanced techniques to achieve complete stone removal and prevent complications (1, 2). While ureteral stones are common in the general population, their management becomes more challenging in patients with comorbidities such as Type 2 Diabetes Mellitus (T2DM). Diabetes contributes to stone formation through alterations in urinary composition, including decreased urinary citrate and increased calcium excretion. Moreover, diabetic patients frequently exhibit impaired renal function, which complicates stone treatment and increases morbidity (3, 4).

Ureteroscopy (URS) is a widely adopted intervention for ureteral stone management, involving the insertion of a ureteroscope into the urinary tract to visualize and remove calculi. There are two primary types of ureteroscopes: rigid and flexible. Rigid ureteroscopy is typically suited for stones in the lower and mid-ureter, while flexible ureteroscopy offers enhanced maneuverability and visualization, particularly for proximal or anatomically challenging stones (5, 6). Each technique has distinct advantages but also notable limitations when used in isolation—especially in complex cases involving multiple or difficult-to-access stones. The combined use of rigid and flexible ureteroscopy has recently gained attention as an effective strategy for managing complex ureteral stones. This hybrid approach leverages the strengths of both modalities, enabling improved access and stone clearance. Such an approach may offer particular benefits in T2DM patients, who face elevated risks of postoperative infection, delayed tissue recovery, and renal impairment (7–9).

Given that T2DM can significantly alter physiological responses to surgical interventions, selecting the most effective and least invasive treatment modality is essential for optimizing outcomes in this population. The combined use of rigid and flexible ureteroscopy may offer a safer and more efficient alternative, minimizing the need for multiple interventions and potentially enhancing recovery. This study aims to evaluate the efficacy of this combined approach in managing complex ureteral stones in patients with T2DM, with a focus on stone clearance rates, postoperative complications, and overall clinical outcomes.

## 2 Methods

### 2.1 Study design

A retrospective cohort study was conducted at our institution between January 2021 and August 2024 to evaluate the efficacy of combined rigid and flexible ureteroscopy in the management of complex ureteral stones in patients with T2DM. Inclusion criteria were age 18–65 years, a confirmed diagnosis of T2DM, and complex ureteral stones—defined as stones >1 cm, multiple stones, or stones located in anatomically challenging sites such as the proximal ureter. All patients had a maximum stone diameter <2 cm and were undergoing their first surgical intervention for stone removal. Exclusion criteria included uncontrolled acute urinary tract infections, distal ureteral obstruction, renal malformations, uncorrectable coagulopathies or bleeding disorders, poor anesthetic or surgical tolerance, active malignancy, concomitant bladder stone requiring complex extraction, and significant psychiatric or communication disorders. A total of 182 patients were enrolled: 93 received combined rigid and flexible ureteroscopy (observation group), and 89 underwent percutaneous nephrolithotomy (PCNL) (control group). The study adhered to the STROBE (Strengthening the Reporting of Observational Studies in Epidemiology) guidelines (10). Informed consent was obtained from all participants. The protocol was reviewed and approved by the institutional ethics committee, with all procedures conducted in accordance with the Declaration of Helsinki. Patient confidentiality was strictly maintained by anonymizing all personal identifiers prior to data analysis.

### 2.2 Preoperative management

Before surgery, patients underwent strict blood glucose control through dietary modifications and appropriate physical activity. Adequate blood glucose control was defined as achieving fasting plasma glucose (FPG) levels between 80–130 mg/dL and HbA1c levels below 7.0% prior to surgery. These thresholds align with commonly accepted guidelines for perioperative glycemic management in diabetic patients, including those from the American Diabetes Association (ADA) (11). For patients with inadequate blood glucose control, insulin therapy was initiated to achieve optimal glucose levels. Surgery was only performed once blood glucose levels were effectively controlled to minimize perioperative complications associated with hyperglycemia, such as infection, delayed wound healing, and poor tissue oxygenation. Additionally, a midstream urine culture was obtained prior to surgery. In cases where the urine culture was positive for bacterial growth, appropriate antimicrobial therapy based on sensitivity testing was administered. Surgery was only conducted after the urine culture became negative, ensuring that urinary tract infections were adequately managed before surgical intervention.

### 2.3 Surgical approach

In the observation group, patients underwent a combined rigid and flexible ureteroscopy procedure under general anesthesia, positioned in the lithotomy position. A rigid ureteroscope (F8.0/9.8, Wolf) was first introduced to access the ureteral stone. Larger calculi were fragmented using a 360 µm holmium laser fiber, and fragments were retrieved with stone forceps. A flexible ureteroscope was then advanced over a guidewire, and a flexible plastic outer sheath (F6.9) was inserted under fluoroscopic guidance. After removing the sheath’s inner core, an Olympus P5 flexible ureteroscope (F6.9) was placed. A 200 µm laser fiber was introduced through the 3.6F working channel, and stone fragmentation was performed in continuous pulse mode at 10–15 W until residual fragments were ≤4 mm in size (12). A double-J stent (F6) was routinely placed at the end of the procedure. Postoperative imaging on day 2 confirmed stent position and assessed stone fragmentation. Stent removal occurred 2–4 weeks postoperatively.

In the control group, patients underwent standard minimally invasive PCNL under general anesthesia. A 6F ureteral catheter was inserted, and patients were positioned prone with the affected kidney elevated. Under ultrasound guidance, percutaneous access was established at the flank between the posterior axillary line and the subscapular angle. A peel-away sheath (F21) was introduced, followed by insertion of a nephroscope (F8.0). Stone fragmentation and extraction were performed using standard PCNL techniques and energy sources tailored to stone composition.

### 2.4 Data collection and outcome measures

Perioperative Indicators: Parameters assessed included intraoperative blood loss, duration of hematuria, length of postoperative hospitalization, and total operative time. In the observation group, operative time was defined from insertion to removal of the combined rigid and flexible ureteroscope. In the control group, it was measured from insertion of the ureteral catheter to removal of the percutaneous nephroscope.

Stone Clearance: Stone clearance was evaluated at 7 days, 1 month, and 3 months postoperatively. Clearance was defined as the absence of radiographic evidence of stones on follow-up abdominal X-rays. Although the 1-month X-ray was used as the primary reference, all time points contributed to the longitudinal assessment of surgical efficacy.

Inflammatory Markers: Inflammatory response was assessed via C-reactive protein (CRP) levels measured preoperatively and on postoperative days 3 and 5. For each assessment, 5 mL of fasting venous blood was collected, centrifuged, and analyzed using enzyme-linked immunosorbent assay (ELISA).

Blood Glucose Levels: Fasting plasma glucose (FPG) and hemoglobin A1c (HbA1c) were monitored preoperatively and on postoperative days 3 and 5. FPG was measured using the glucose oxidase method from 3 mL of fasting venous blood. HbA1c was quantified via ion-exchange high-performance liquid chromatography (HPLC) from 2 mL of anticoagulated venous blood.

Safety and Complications (13): Postoperative sepsis occurring within two weeks was used as the primary safety endpoint. Multivariate analysis was conducted to identify risk factors associated with sepsis, including perioperative glycemic control, inflammatory markers, and stone-related characteristics.

No auxiliary procedures (secondary URS, ESWL, or repeat PCNL) were undertaken during the 3-month follow-up; stone clearance rates at 1 and 3 months reflect the clinical course after the initial intervention without additional lithotripsy.

### 2.5 Statistical analysis

Statistical analyses were performed using IBM SPSS Statistics (Version 27.0, IBM Corp., Armonk, NY, USA). Continuous variables were presented as mean ± standard deviation (SD) or median (interquartile range), as appropriate. Normality and homogeneity of variances were assessed using the Shapiro–Wilk test and Levene’s test, respectively. Variables meeting assumptions were compared using independent-sample t-tests; variables violating these assumptions were analyzed using Welch’s t-test or the Mann–Whitney U test. Categorical variables were summarized as frequencies (percentages) and compared using the Chi-square (χ²) test or Fisher’s exact test when appropriate. Effect sizes (Cohen’s d for continuous variables and Cohen’s h for categorical variables) with 95% confidence intervals (CIs) were calculated to complement p-values. Multivariate logistic regression analysis was conducted using a backward stepwise approach to identify independent predictors of sepsis among variables with a univariate p-value < 0.20. Multicollinearity was evaluated by variance-inflation factor (VIF, acceptable if < 5), model fit by Hosmer–Lemeshow goodness-of-fit test, and discriminative performance assessed by the area under the receiver operating characteristic (ROC) curve (AUC).

## 3 Results

### 3.1 General characteristics comparison between the observation and control groups

Baseline characteristics were well balanced between the observation group (n = 93) and the control group (n = 89). No statistically significant differences were observed in sex distribution (P = 0.341), stone laterality (P = 0.559), or age (P = 0.616). Other parameters, including duration of diabetes (P = 0.337), duration of stone disease (P = 0.295), BMI (P = 0.812), number of stones (P = 0.228), and maximum stone length (P = 0.801), were also comparable. In terms of stone characteristics, no significant differences were found between groups in terms of stone segment distribution (upper, middle, lower ureter, UVJ, or multi-segmental involvement), stone density measured in Hounsfield units (P = 0.720), or serum creatinine levels (P = 0.483). The degree of hydronephrosis was similar between the two groups (P = 0.591), with mild to moderate hydronephrosis predominating in both. These findings confirm that the two cohorts were appropriately matched with respect to demographic, anatomical, and radiological variables, thereby ensuring comparability for outcome analysis (**Table 1**).

**Table 1:** Comparison of general characteristics between the observation and control groups in T2DM patients with complex ureteral stones.

Characteristic	Observation group (n=93)	Control group (n=89)	Statistics (t/χ2)	P-value
Gender [n (%)]	-	-	0.906	0.341
Male	42 (45.16%)	34 (38.20%)	–	–
Female	51 (54.84%)	55 (61.80%)	–	–
Stone Location [n (%)]	-	-	0.341	0.559
Left Side	43 (46.24%)	45 (50.56%)	–	–
Right Side	50 (53.76%)	44 (49.44%)	–	–
Stone Segment [n (%)]				
Upper Ureter (UUS)	20 (21.51%)	22 (24.72%)		
Middle Ureter (MUS)	15 (16.12%)	13 (14.61%)		
Lower Ureter (LUS)	40 (43.01%)	38 (42.70%)		
UVJ (Ureterovesical Junction)	12 (12.90%)	10 (11.24%)		
Multi-segmental (Entire Ureter)	6 (6.45%)	6 (6.74%)		
Age (x ± s, years)	56.25 ± 5.85	55.80 ± 6.22	0.503	0.616
Duration of Diabetes (x ± s, years)	7.75 ± 2.05	8.05 ± 2.15	0.964	0.337
Duration of Stone Disease (x ± s, months)	8.12 ± 1.13	7.94 ± 1.18	1.051	0.295
BMI (x ± s, kg/m²)	24.06 ± 3.15	23.95 ± 3.08	0.238	0.812
Number of Stones (x ± s, stones)	4.08 ± 0.68	4.21 ± 0.77	1.209	0.228
Stone Length (x ± s, cm)	1.39 ± 0.50	1.37 ± 0.57	0.252	0.801
Stone density (HU), mean ± SD	952 ± 148	944 ± 153	0.359	0.720
Hydronephrosis severity, n (%)			1.051	0.591
– Mild	39 (41.94%)	37 (41.57%)		
– Moderate	32 (34.41%)	30 (33.71%)		
– Severe	22 (23.66%)	22 (24.72%)		
Serum creatinine (µmol/L), mean ± SD	85.3 ± 17.6	87.1 ± 16.9	0.703	0.483

BMI, Body Mass Index.

T2DM, Type 2 Diabetes Mellitus.

### 3.2 Comparison of perioperative parameters between the observation and control groups

Significant differences in perioperative parameters were observed between the observation and control groups. The control group had a shorter surgery time (86.72 ± 19.87 min vs. 64.84 ± 14.88 min, P<0.001) and less intraoperative blood loss (16.02 ± 4.89 mL vs. 45.95 ± 9.87 mL, P<0.001). Additionally, the duration of hematuria was shorter in the observation group (2.58 ± 0.31 days vs. 4.41 ± 0.73 days, P<0.001). The observation group also had a shorter postoperative hospitalization (3.68 ± 0.48 days vs. 5.50 ± 0.72 days, P<0.001), indicating more favorable perioperative outcomes (**Table 2**).

**Table 2:** Comparison of perioperative parameters between the observation and control groups in T2DM patients with complex ureteral stones (Mean ± SD).

Parameter	Observation group (n=93)	Control group (n=89)	t-value	p-value
Surgery Time (min)	86.72 ± 19.87	64.84 ± 14.88	8.380	<0.001
Intraoperative Blood Loss (mL)	16.02 ± 4.89	45.95 ± 9.87	26.09	<0.001
Hematuria Duration (days)	2.58 ± 0.31	4.41 ± 0.73	22.18	<0.001
Postoperative Hospitalization (days)	3.68 ± 0.48	5.50 ± 0.72	20.15	<0.001

T2DM, Type 2 Diabetes Mellitus.

### 3.3 Comparison of stone clearance rates between the observation and control groups

The comparison of stone clearance rates between the observation group (combined rigid and flexible ureteroscopy treatment) and the control group (percutaneous nephrolithotomy) revealed notable differences. At 7 days postoperatively, the observation group demonstrated a significantly higher stone clearance rate (54.84%) compared to the control group (39.33%) (P=0.036). However, at 1 month and 3 months postoperatively, the differences between the two groups were less pronounced. At 1 month, the observation group had a clearance rate of 94.62%, while the control group had 89.89% (P=0.231). By 3 months, the stone clearance rate in the observation group was 100%, slightly higher than the control group’s 96.63%, but the difference was not statistically significant (P=0.074) (**Table 3**). The improvement in clearance from day 7 to months 1 and 3 resulted from spontaneous passage of residual fragments under stent drainage and from fragment retrieval at routine stent removal; no auxiliary URS, ESWL, or repeat PCNL was undertaken during the follow-up period. These findings suggest that combined ureteroscopy treatment may offer superior early stone clearance compared to percutaneous nephrolithotomy in T2DM patients with complex ureteral stones.

**Table 3:** Comparison of stone clearance rates between the observation and control groups in T2DM patients with complex ureteral stones [n (%)].

Parameter	Observation group (n=93)	Control group (n=89)	χ²	p-value
Stone Clearance Rate Postoperative 7 Days	51 (54.84%)	35 (39.33%)	4.391	0.036
Stone Clearance Rate Postoperative 1 Month	88 (94.62%)	80 (89.89%)	1.437	0.231
Stone Clearance Rate Postoperative 3 Months	93 (100.00%)	86 (96.63%)	3.187	0.074

T2DM, Type 2 Diabetes Mellitus.

### 3.4 Comparison of CRP levels between the observation and control groups

Preoperative CRP levels were similar between the two groups, with no significant difference (P=0.791). However, at 3 days postoperatively, the observation group had significantly lower CRP levels (17.98 ± 1.57 mg/L) compared to the control group (19.87 ± 1.72 mg/L, P<0.001). This trend persisted at 5 days postoperatively, with CRP levels remaining lower in the observation group (9.16 ± 1.79 mg/L vs. 10.12 ± 2.04 mg/L, P<0.001) (**Table 4**). These results highlight the reduced inflammatory response associated with combined ureteroscopy treatment.

**Table 4:** Comparison of CRP levels between the observation and control groups in T2DM patients with complex ureteral stones (Mean ± SD).

CRP (mg/L)	Observation group (n=93)	Control group (n=89)	t-value	p-value
Preoperative	15.14 ± 1.88	15.21 ± 1.66	0.266	0.791
Postoperative 3 Days	17.98 ± 1.57	19.87 ± 1.72	7.748	<0.001
Postoperative 5 Days	9.16 ± 1.79	10.12 ± 2.04	3.378	<0.001

CRP, C-reactive protein.

T2DM, Type 2 Diabetes Mellitus.

### 3.5 Univariate analysis of general data related to sepsis in patients with T2DM complicated by complex ureteral stones post-surgery

The univariate analysis revealed key factors related to the development of sepsis in T2DM patients following surgery for complex ureteral stones. Significant associations were observed with gender, age, BMI, and certain biochemical markers. Specifically, females (83.33%) were more likely to develop sepsis than males (16.67%) (P=0.026). Older patients (≥60 years) had a higher incidence of sepsis (72.22%) compared to younger patients (<60 years) (27.78%) (P=0.025). Similarly, patients with a BMI ≥25 kg/m² had a significantly higher risk of sepsis (77.78%) than those with a BMI <25 kg/m² (22.22%) (P=0.005). In terms of biochemical markers, higher postoperative levels of CRP, FPG, and HbA1c were associated with sepsis development. CRP levels on postoperative day 3 were significantly elevated in the sepsis group (P<0.001), and a similar trend was observed on postoperative day 5 (P<0.001). Furthermore, postoperative FPG levels were higher in the sepsis group compared to the non-sepsis group (P<0.001), indicating poor glycemic control as a contributing factor. Additionally, HbA1c levels were also significantly higher in the sepsis group (P=0.025), suggesting that poor long-term glycemic management may increase the risk of sepsis. No significant differences were found in the duration of diabetes, stone duration, number of stones, maximum stone diameter, or hydronephrosis degree between the two groups. These findings highlight that gender, age, BMI, and biochemical markers, particularly CRP, FPG, and HbA1c, are critical factors for sepsis development in these patients (**Table 5**).

**Table 5:** Univariate analysis of general data related to sepsis in patients with T2DM complicated by complex ureteral stones post-surgery.

Parameter	Sepsis group (n=18)	Non-sepsis group (n=164)	t/χ²	P Value
Gender [n (%)]			4.966	0.026
Male	3 (16.67%)	72 (43.90%)		
Female	15 (83.33%)	92 (56.10%)		
Age [n (%)]			4.997	0.025
<60 years	5 (27.78%)	91 (55.49%)		
≥60 years	13 (72.22%)	73 (44.51%)		
BMI [n (%)]			8.038	0.005
<25 kg/m²	4 (22.22%)	94 (57.32%)		
≥25 kg/m²	14 (77.78%)	70 (42.68%)		
Diabetes Duration (x ± s, years)	8.14 ± 2.35	7.72 ± 2.10	0.796	0.427
Stone Duration (x ± s, months)	7.95 ± 1.12	7.85 ± 1.07	0.375	0.708
Number of Stones (x ± s)	4.28 ± 0.69	4.05 ± 0.64	1.436	0.153
Max Stone Diameter (x ± s, cm)	1.52 ± 0.28	1.43 ± 0.32	1.145	0.254
Hydronephrosis Degree [n (%)]			0.008	0.996
Mild	7 (38.89%)	69 (42.07%)		
Moderate	6 (33.33%)	56 (34.15%)		
Severe	5 (27.78%)	39 (23.78%)		
Preoperative Urine Culture Positive [n (%)]	7 (38.89%)	18 (10.98%)	10.67	0.001
Surgical Method [n (%)]			1.192	0.275
Ureteroscopic Combined Surgery	7 (38.89%)	86 (52.44%)		
Minimally Invasive Percutaneous Nephrolithotomy	11 (61.11%)	78 (47.56%)		
Surgical Duration (x ± s, min)	78.12 ± 20.46	75.17 ± 18.52	0.635	0.526
CRP (x ± s, mg/L)				
Preoperative	15.19 ± 1.78	15.26 ± 1.85	0.153	0.879
3 Days Postoperatively	29.41 ± 1.89	15.79 ± 1.65	32.76	<0.001
5 Days Postoperatively	25.42 ± 2.41	8.11 ± 1.12	53.71	<0.001
Postoperative FPG (x ± s, mg/dL)	13.00 ± 2.11	5.06 ± 1.32	22.62	<0.001
HbA1c (x ± s, %)	8.56 ± 0.41	5.02 ± 0.39	36.38	<0.001

CRP, C-Reactive Protein.

FPG, Fasting Plasma Glucose.

HbA1c, Hemoglobin A1c.

### 3.6 multivariate logistic regression analysis of risk factors for postoperative sepsis in T2DM patients with complex ureteral stones

Multivariate logistic regression analysis was conducted to identify independent risk factors associated with postoperative sepsis in patients with T2DM and complex ureteral stones. The analysis revealed several significant factors, all of which were strongly associated with an increased risk of sepsis. Female gender (OR = 5.71, 95% CI: 1.282-25.184, P < 0.001) was found to be an independent risk factor for sepsis. Additionally, older age (≥60 years) was also a significant predictor (OR = 6.406, 95% CI: 1.308-33.493, P < 0.001). Elevated BMI (≥25 kg/m²) further increased the likelihood of sepsis (OR = 6.016, 95% CI: 1.287-31.378, P < 0.001). Moreover, preoperative positive urine cultures (OR = 7.804, 95% CI: 1.552-45.567, P < 0.001) were strongly associated with the occurrence of sepsis. Postoperative CRP levels on both days 3 (OR = 8.246, 95% CI: 1.564-49.302, P < 0.001) and 5 (OR = 5.268, 95% CI: 1.035-28.124, P < 0.001) were also significant indicators of sepsis risk. Similarly, postoperative FPG levels (OR = 8.506, 95% CI: 1.608-48.239, P < 0.001) and HbA1c levels (OR = 5.529, 95% CI: 1.086-29.644, P < 0.001) were found to significantly increase the risk of sepsis. These findings suggest that factors such as female gender, advanced age, higher BMI, preoperative positive urine culture, as well as postoperative inflammatory and glycemic markers (CRP, FPG, and HbA1c), play a crucial role in predicting postoperative sepsis in T2DM patients undergoing surgery for complex ureteral stones (**Table 6**).

**Table 6:** Multivariate logistic regression analysis of risk factors for postoperative sepsis in T2DM patients with complex ureteral stones.

Factor	Regression coefficient	Standard error	Wald χ²	Odds ratio (OR)	95% confidence interval (CI)	P value
Gender (Female)	1.732	0.465	13.967	5.71	1.282, 25.184	< 0.001
Age ≥ 60 years	1.854	0.478	14.975	6.406	1.308, 33.493	< 0.001
BMI ≥ 25 kg/m²	1.798	0.389	21.546	6.016	1.287, 31.378	< 0.001
Preoperative Urine Culture Positive	2.052	0.387	28.476	7.804	1.552, 45.567	< 0.001
CRP Postoperative 3 Days	2.107	0.495	19.036	8.246	1.564, 49.302	< 0.001
CRP Postoperative 5 Days	1.662	0.471	12.332	5.268	1.035, 28.124	< 0.001
Postoperative FPG	2.14	0.473	20.592	8.506	1.608, 48.239	< 0.001
HbA1c	1.708	0.487	13.321	5.529	1.086, 29.644	< 0.001

BMI, Body Mass Index.

CRP, C-reactive Protein.

FPG, Fasting Plasma Glucose.

HbA1c, Hemoglobin A1c.

### 3.7 *Post hoc* power analysis

A weighted *post hoc* power analysis was conducted to assess the overall statistical power of the study based on key clinical outcomes. The primary endpoint—stone clearance rate at postoperative day 7—was used as the principal variable, supplemented by critical secondary outcomes, including operative time, intraoperative blood loss, and CRP levels on postoperative day 3, all of which showed statistically significant intergroup differences. Each variable was assigned a clinical weight reflecting its importance in evaluating surgical efficacy and perioperative safety. Using a two-sided α level of 0.05 and observed effect sizes from our data, the weighted composite power was calculated to be 82% (>80%), indicating adequate sensitivity to detect clinically meaningful differences between the two surgical modalities. This *post hoc* evaluation supports the robustness of our sample size and reinforces the validity of the study’s conclusions.

## 4 Discussion

This study provides novel insights into the management of complex ureteral stones in patients with T2DM, a population at high risk for perioperative complications. The comparison of combined rigid and flexible ureteroscopy (RIRS+URS) with PCNL in this cohort is clinically significant, as it sheds light on the advantages of RIRS+URS in terms of early stone clearance, reduced postoperative inflammation, and shorter recovery times. The study’s innovative aspect lies in its focus on glycemic control, inflammatory markers (CRP), and their role in predicting postoperative sepsis, a critical concern in T2DM patients. Moreover, the inclusion of detailed perioperative parameters such as stone density, hydronephrosis severity, and preoperative creatinine levels adds depth to the understanding of how these factors influence treatment outcomes. Clinically, this study emphasizes the need for individualized treatment strategies, incorporating both surgical choices and metabolic control, to optimize patient outcomes. The results suggest that RIRS+URS may offer superior early outcomes compared to PCNL, with reduced risks of sepsis and faster recovery, particularly in T2DM patients. The identification of key risk factors—such as gender, age, BMI, and glycemic control markers—provides valuable guidance for clinicians in predicting and mitigating complications, thus improving perioperative management strategies (14–16). This work contributes to advancing the field of endourology by offering evidence-based recommendations for the management of complex ureteral stones in T2DM patients, with potential for broader clinical application in similar high-risk patient populations.

The comparison of perioperative parameters between the observation group (combined rigid and flexible ureteroscopy) and the control group (percutaneous nephrolithotomy) revealed significant differences in several key measures, including surgery time, intraoperative blood loss, hematuria duration, and postoperative hospitalization. The observation group exhibited notably shorter surgery times and less intraoperative blood loss, in addition to a significantly shorter duration of hematuria and reduced postoperative hospitalization. These findings suggest that the combined ureteroscopy approach is associated with less tissue trauma, fewer complications, and quicker recovery compared to percutaneous nephrolithotomy. The higher stone clearance rate at 7 days in the observation group (54.84%) compared to the control group (39.33%) underscores the benefit of combined ureteroscopy in achieving faster stone removal in the early postoperative period. However, by 1 month and 3 months post-surgery, both groups exhibited similar high stone clearance rates, indicating that while the combined ureteroscopy technique offers superior early outcomes, both methods ultimately yield comparable long-term success in terms of stone clearance. These results suggest that the choice of surgical technique may impact the early postoperative course, but the long-term effectiveness of both treatments in achieving stone clearance remains similar (17, 18).

Sepsis remains a critical concern in surgical outcomes, particularly in high-risk populations such as T2DM patients with complex ureteral stones (19, 20). Our multivariate logistic regression analysis identified several key risk factors for postoperative sepsis, including female gender, age ≥60 years, BMI ≥25 kg/m², preoperative positive urine culture, and elevated postoperative levels of inflammatory markers (CRP, FPG, and HbA1c). These findings are consistent with existing literature, highlighting that female patients are more prone to infections due to hormonal differences and a higher incidence of urinary tract infections (UTIs) (21, 22). Age ≥60 years, a well-established risk factor, is often associated with immune decline and increased comorbidities, such as cardiovascular disease and renal dysfunction, which complicate recovery and elevate sepsis risk. Obesity, reflected by BMI ≥25 kg/m², was also associated with a heightened sepsis risk, likely due to systemic inflammation, impaired immune function, and reduced wound healing (23, 24). Preoperative positive urine culture further emphasized the importance of addressing UTIs before surgery to reduce infection-related complications. Additionally, elevated CRP, FPG, and HbA1c levels postoperatively were strong predictors of sepsis, reinforcing the role of systemic inflammation and poor glycemic control in infection susceptibility (25, 26). These findings underscore the importance of early identification of high-risk patients and tailored interventions, including optimized glycemic control, antibiotic management, and post-surgical monitoring, to mitigate sepsis risk and improve surgical outcomes in this vulnerable population.

Integrating findings from recent studies enhances the contextual understanding of our results. Castellani et al. (27) demonstrated that diabetes and positive stone culture were significant predictors of sepsis following flexible ureteroscopy. Similarly, our findings identified T2DM-related factors—such as elevated CRP, FPG, and BMI—as independent sepsis risk predictors. Unlike their multicenter prospective study focusing on renal stones, our study expands the evidence by targeting complex ureteral stones and comparing surgical modalities, thereby offering novel insight into procedure-specific risks in T2DM patients. Kino et al. (21)highlighted that older age, female sex, diabetes, and poor performance status were associated with preoperative urosepsis. Consistent with these findings, our study identified age ≥60, female gender, and obesity as significant predictors of postoperative sepsis. While their study focused on preoperative infectious risk, our analysis complements this by elucidating modifiable postoperative predictors, emphasizing the importance of perioperative optimization in T2DM patients. Chou et al. (28) reported that a hybrid laparoscopic and endoscopic approach achieved favorable stone clearance and low sepsis rates in a diabetic patient. In contrast, our cohort study quantitatively demonstrates that combined rigid and flexible ureteroscopy yields superior early stone-free rates and reduced inflammation compared to PCNL in T2DM patients with ureteral, not renal, stones—offering broader clinical applicability and procedural efficiency.

This study has several limitations. First, its retrospective and non-randomized design introduces potential selection bias and limits causal inference. Although the sample size was adequate for statistical comparisons, it may not reflect the full clinical heterogeneity of the broader T2DM population, particularly in terms of comorbidities and stone characteristics. Second, stone composition data were not consistently available due to incomplete stone retrieval, limiting our ability to explore associations between stone type and infection risk. Third, the definition of stone-free status as residual fragments <4 mm may underestimate infection risk in T2DM patients, who are more susceptible to complications even from small residual fragments. Fourth, stone clearance was evaluated using KUB radiography, which has limited sensitivity for detecting fragments <4 mm. KUB was selected for its lower radiation exposure and compatibility with institutional reimbursement policies, but future studies should utilize NCCT for more accurate assessment. Lastly, while sepsis was a key endpoint, other infectious complications such as SIRS and febrile episodes were not included. Future prospective, multicenter randomized trials with standardized imaging and broader outcome measures are needed to validate and expand upon these findings.

## 5 Conclusions

In conclusion, combined rigid and flexible ureteroscopy was associated with more favorable perioperative outcomes and higher early stone clearance rates compared to percutaneous nephrolithotomy in patients with T2DM and complex ureteral stones. This approach was linked to shorter operative time, reduced blood loss, faster recovery, and lower postoperative inflammatory response as indicated by CRP levels. Additionally, the analysis identified several factors associated with an increased risk of postoperative sepsis, including advanced age, female sex, elevated BMI, positive preoperative urine culture, and higher postoperative CRP and FPG levels. These findings underscore the importance of individualized perioperative management and risk stratification in this high-risk population. Further prospective studies are warranted to assess long-term outcomes, recurrence patterns, and the effectiveness of sepsis prevention strategies.

## Data Availability

The raw data supporting the conclusions of this article will be made available by the authors, without undue reservation.
